# Learning from orthopaedic complications and adverse events in a district general hospital

**DOI:** 10.1308/rcsann.2025.0064

**Published:** 2026-01-12

**Authors:** AP Dekker, A Stammer, N Ashwood

**Affiliations:** ^1^ Royal Derby Hospital, UK; ^2^ Queens Hospital, UK; ^3^ Burton Hospitals NHS Foundation Trust, UK

**Keywords:** Complications, Adverse events, Mortality, Surgical experience

## Abstract

**Introduction:**

The aim of this study was to determine the learning derived from postoperative complications to reduce the risk of avoidable harm.

**Methods:**

A retrospective review of a prospectively maintained local hospital registry of readmissions for complications was undertaken at a single district general hospital over a 10-year period (November 2014–November 2024). Learning points policy changes were monitored prospectively using audit to determine influence on clinical practice.

**Results:**

There were 316 elective complications from 22,658 cases (1.4%) and 251 trauma complications from 15,862 cases (1.6%). There were 293 deaths out of the 38,520 total cases (0.76% mortality rate); of these, 16 occurred in elective surgery (0.07% mortality rate) and 241 trauma cases out of 15,852 (rate of 1.6%). The total number of elective cases requiring reoperation was 374. The cases from different hospitals accounted for 41%; there were 251 local trauma cases that required reoperation. The most frequent complication was implant dislocation in 201 cases, which was avoidable in one-third of cases due to technical issues. Most periprosthetic fractures were a late complication secondary to osteoporosis. Discussion of the care of complex cases enabled improved training packages and pathways to manage wound care, infection and the technical aspects of prosthetic joint implantation and fracture fixation for trainee surgeons.

**Conclusions:**

There is a significant resource implication for managing complications that originate from other hospitals. Monitoring complications and mortality helps improve practice and enable reproducible outcomes.

## Introduction

The Royal College of Surgeons of England has guidelines for professional conduct and reflection on learning from mortality and morbidity meetings.^1^ Although meetings are undertaken in trauma and orthopaedic departments in hospital trusts in the UK, to our knowledge there is no published literature concerning what was learned or how best to structure a meeting to facilitate learning institutionally and disseminate to a wider audience. There is educational value for surgeons in training and peers in sharing experiences and learning from encountered unexpected complications and patient outcomes.^2^ Surgical skills are complex and take time to acquire, even for expert surgeons and for trainees, with learning curves described.^3^ Steps such as mentoring for individual trainees can be taken to mitigate risks, but group learning is also valuable and necessary.^3–5^ Vigilance and a recognition that ‘to err is human’ helps us foster a learning culture where discussion of results can allow the wider team to hopefully avoid common pitfalls and allow service development as changes occur in the dynamic, ever-changing, health service where personnel turnover is high and innovation is constant.^6^

Most units conduct regular reviews of mortality and morbidity. However, this is often in isolation and often nonstructured or disorganised. RCS England recommends using the Situation, Background, Assessment and Recommendation (SBAR) system,^1^ grading harm and recording further recommendation using the National Confidential Enquiry into Patient Outcome and Death (NCEPOD).^7^ Learning points should be fed back to influence trust guidelines, and documented as part of local incident reporting policy, professional duty of candour and learning through safety meetings. It is these latter processes of dissemination within a unit, trust and nationally that the authors feel need strengthening. No national registry exists to record similar complications in a standardised format; neither are there guidelines for thematic analysis of core complications and top tips for how to avoid them. This is particularly important in surgery as a discipline, as techniques and equipment evolve and innovation occurs constantly, and our first aim as surgeons is to ‘reduce any further harm’. The tendency is for data on complications and adverse events to be used to rank the performance of a unit rather than to inform practice and embed learning.

The aim of this study was to determine the learning derived from postoperative complications by better understanding the pattern of presentation and care delivery to guide treatment pathways and training to reduce the risk of avoidable harm.

## Methods

A retrospective review of a prospectively maintained local hospital registry of readmissions for complications was undertaken at a single district general hospital over a 10-year period (November 2014–November 2024). This included a thematic analysis to define the categories of common learning points from the minutes of the monthly mortality and morbidity meetings for patients requiring readmission and subsequent policy and pathway changes that altered clinical practice.

Data, including patient demographic details, comorbidities classified using the Charlson Comorbidity Index as updated by Quan *et al*,^8,9^ details of the complication, subsequent shared learning and identified avoidability of harm, were recorded.

A complication was defined as any deviation from the normal expected postoperative state.^10^ Expected consequences of surgery or treatment failure due to disease recurrence have been excluded in line with previous studies.^11^ Complications were classified as avoidable or unavoidable at the multidisciplinary team (MDT) meeting on the basis that if different actions were undertaken then the outcome may have been different, and the readmission avoided including further surgical or medical treatment such as intravenous antibiotic therapy. Reoperation was defined as a return to the operating theatre for further surgical treatment in relation to the original pathology. Avoidable complications included wound infections or collections, technical failure and metabolic disturbance due to poor fluid management in renally impaired patients. Unavoidable complications included allergic reaction, failure related to further trauma, deep vein thrombosis despite appropriate thromboprophylatic measures, cardiovascular complication (cerebrovascular accident, myocardial infarction) or wear-related complications. Complications were classified for level of harm by the MDT (including at least one consultant) during the morbidity and mortality meeting according to the Clavien–Dindo Classification (1 = no pharmacological, surgical, endoscopic or radiological intervention; 2 = pharmacological treatment including blood transfusions; 3 = surgical, endoscopic or radiological intervention; 4 = life-threatening organ dysfunction requiring intensive care treatment; 5 = death).^12^ Recommended learning points or actions were recorded by the MDT. Thematic analysis was further derived from analysis of the MDT reports for the purpose of this study.

Descriptive statistical analysis was performed using Statistical Package for the Social Sciences (SPSS®, version 23.0; SPSS Inc, Chicago, IL, US).

## Results

There were 777 complications over 10 years; over the same time period, there were 38,520 procedures, equating to a rate of 2%. However, from this group, 0.55% had their original procedure performed in other hospitals but presented to our local unit (rather than being referred), accounting for 27% of total complications. The local hospital complication rate was therefore 1.45%. Some patients had the same complication more than once; for example, multiple dislocations. These multiple events affected 43 patients. For the purposes of considering the impact of each event, we have kept these as separate complications in this stage of the analysis.

From our centre, there were 316 elective complications from 22,658 cases (1.4%) and 251 trauma complications from 15,862 cases (1.6%).

Regarding reoperations at our institution, the reoperation rate in elective cases was 222 cases out of the 22,658 complications (0.9%). Most of these were for wound infections in 74 cases (21 hip, 23 knee, 10 shoulder, 25 other), with evacuation of haematoma in 40 cases (24 hip, 11 knee, 4 other) in the presence of infection or threatened skin. In the arthroplasty cases, 71 periprosthetic fractures required fixation (31 hip, 15 knee, 5 shoulder) or revision (14 hip and 6 knee revision arthroplasties) and 37 arthroplasties were revised either for infection (16 for hip infection, 5 for knee and 3 for shoulder infection) or instability (13 for hip instability). The total number of cases for each joint replacement is shown in Table 1.

**Table 1:** Local complication rate (including wound problems) by prosthesis

Procedure	Total complications	Total cases	Rate
Knee replacement	38	3,570	1%
Hip replacement	73	3,245	2.3%
Shoulder replacement	8	339	2.4%

Regarding reoperations from patients who had their primary surgery in other hospitals, there were 152 cases that required surgical treatment; 42 total hip replacement (THR) dislocations all needed surgery, with 24 revisions due to wear. There were 30 periprosthetic fractures, 8 cases of wound infection that were debrided and 35 cases of infected prosthesis (22 total knee replacement (TKR) and 13 THR).

The total number of elective cases requiring reoperation was 374. The cases from different hospitals accounted for 41%.

In the wound problems from imported cases, 12 wound problems happened in trauma cases. No major trauma complications occurred in this group.

Of the 251 local trauma complications, there were 71 cases of failure of fixation (28%); these were hip and ankle fixation failures mainly, which required revision of fixation. Infection and wound problems accounted for 69 cases (27.5%). The distribution of the area of the complication is shown in Table 2.

**Table 2:** Distribution complication by area

Trauma	N	Total cases	Rate
Hip hemiarthroplasty or THR	1,800	64	3.6%
DHS	1,200	28	2.3%
Intramedullary nailing	412	15	3.6%
Foot and ankle cases	1,975	29	1.5%
Knee and tibia	2,127	10	0.5%
Upper limb	4,905	44	0.9%

THR = total hip replacement; DHS = dynamic hip screw.

There were 293 deaths in the 38,520 total local cases (0.76% local mortality rate); 16 occurred in elective surgery (0.07% mortality rate), 6 admissions had periprosthetic infection, 6 had periprosthetic fracture, 3 had hip dislocation and 1 had sepsis in an elective TKR.

The mortality rate in trauma patients was 241 cases out of 15,852 (rate of 1.6%). There were 211 hip fragility fractures accounting for 0.8% of the mortality. The timing of the mortality following the index operation is shown in Table 3.

**Table 3:** In hospital mortality

	Immediate mortality	Early <6 weeks mortality
Hip	205	6
Not hip	28	2

There were an additional 95 deaths, 8 in elective patients at 36 months (0–240 months) and 87 in trauma patients after the revision surgery at 10 months median (range 0–60 months).

When considering patient factors contributing to complication rates, there were 179 smokers (23%). Age was median 81 years (range 38–97) in 342 male and 732 female patients. Patients had a median of two comorbidities (range 0–10) with a Charlson updated score of median 0 (range 0–5).

Clavien–Dindo classification of level of harm was median 3 (Table 4).^10^ From the level 5 harm cases, the cause of death was bronchopneumonia, cardiac or frailty problems in all cases. In 30 cases, recommendations that improved protocols and guidelines were put in place.

**Table 4:** Level of harm of complication as classified by Clavien and Dindo

Harm	Frequency	Rate
1	15	1%
2	198	18%
3	558	50%
4	3	<1%
5	336	30%

When assessing the level of harm grade 1–4, the frequency of complications excluding mortality is listed in Table 5.

**Table 5:** Frequency of complications for levels of harm excluding mortality

Complication themes	Frequency	
Acute implant instability/dislocation	201	27%
Periprosthetic fracture	92	12%
Implant infection	88	12%
Wound infection or dehiscence	132	18%
Fixation failure	66	9%
Infection (chest/urine)	37	5%
Haematoma	36	5%
DVT	12	2%
Joint stiffness	21	3%
Pain	14	2%
Compartment syndrome	10	1%
Pressure ulcer	5	1%
Pulmonary embolism	5	1%
Nerve injury	7	1%
Vascular injury	8	1%
Cardiac arrest	3	<1%
Stroke	3	<1%
Metabolic problem	3	<1%
Confusion	2	<1%
Tendon rupture	2	<1%
Total	747	

DVT = deep vein thrombosis.

A total of 428 complications had an element of avoidable harm in the following areas of patient care, whereas 346 were considered unavoidable. The themes are summarised in Table 6. Of note, there were elements of surgical technique that could be improved in 115 cases (27%), infection management in 154 cases (36%), wound care in 118 cases (28%) and fracture management in 92 cases (21%).

**Table 6:** Complication themes

Theme	Frequency
Surgical technique	115
Infection management	154
Wound care	118
Fracture management	92
Implant wear	71
Patient selection	46
Vascular injury or management of bleeding	33
Awareness	21
Neurological injury	8
Metabolic disturbance	14

The rates of complications relating to hip arthroplasty, knee arthroplasty, shoulder arthroplasty, hip fracture fixation and ankle fracture fixation are shown in Tables 7–11, respectively.

**Table 7:** Hip arthroplasty complications

	Frequency	%	Literature^13^
Infection	16	0.5%	1%
Instability	13	0.4%	1.5%
Periprosthetic fracture	45	1.4%	0.6–1%
Total cases	3,245		

**Table 8:** Knee arthroplasty complications

	Frequency	%	Literature^14,15^
Infection	5	0.1%	0.6%
Instability	0	0%	0%
Periprosthetic fracture	21	0.6%	0.3–5.5%
Total cases	3,570		

**Table 9:** Shoulder arthroplasty complications

	Frequency	%	Literature^16–20^
Infection	3	0.9%	1–2.5%
Instability	6	1.8%	1–2.2%
Periprosthetic fracture	5	1.5%	1.5%
Total cases	339		

**Table 10:** Hip fracture complications

	Frequency	%	Literature^21–23^
Cut out DHS	30	2.6%	1.5–2.6%
Superficial wound infection	28	0.9%	1–5%
Deep infection	15	0.5%	1–2%
Instability	13	0.7%	2–4%
Total complications	96		
Total DHS cases	1,200		
Total hip hemiarthroplasty/THR cases	1,800		

THR = total hip replacement; DHS = dynamic hip screw.

**Table 11:** Ankle fracture complications

	Frequency	%	Literature^21,24,25^
Fixation failure	10	0.9%	1–3%
Superficial wound infection	12	1.1%	1–6%
Deep infection	12	1.1%	1–9%
Instability	5	0.5%	1%
Total complications	39		

## Discussion

This study has identified common complications from patients undergoing orthopaedic surgical treatment. The most frequent complications were related to implant instability and dislocation acutely in 130 cases where the arthroplasty surgeons in the unit felt that the acetabular cup placement or femoral stem version was suboptimal. Although hip stability is multifactorial, acetabular component orientation and position are important factors in reducing the risk of dislocation and, in general, it is agreed that inclination angles greater than 50° or excessive anteversion with respect to the transverse acetabular ligament should be avoided.^26^ Further training was undertaken to target implant placement by the senior surgeons.

In 71 cases, the complication was felt to be a consequence of implant wear and therefore not avoidable. These 71 cases were detected over a 10-year period; however, the index operations took place during the preceding 15- to 35-year timespan. The reported complications therefore reflect the observed prevalence over that period. The number of arthroplasties performed during the study period was 20,000, a proportion similar to literature values. The National Joint Registry 2023 reported an aseptic loosening rate of 1 per 1,000 prosthesis-years for hip and knee replacements.^27^

Very few periprosthetic fractures occurred acutely (within 6 weeks of the index procedure) (6 of 92 cases) and typically had a mechanism of injury of fall from standing height or less (fragility fractures); this was felt to relate more to osteoporosis and therefore pathways were developed to reinforce fragility fracture care in the unit. The patients were also seen by an orthogeriatric consultant physician (Care of the Elderly team) and started on osteoporotic medications as part of the fragility fracture review process. Periprosthetic fractures were presented in the general meeting for review and group learning but also escalated to the hip, knee and shoulder MDTs for further subspecialty discussion.

Wound problems, which included infection, dehiscence and local haematoma, were also frequent, occurring in 168 cases. Approximately one-third (56 cases) were felt to be unavoidable because of patient compliance with postoperative instructions and the need for anticoagulation for the prevention of venous thromboembolism. In the remaining cases, better compliance with antibiotic regimens and haemostasis during wound closure was felt to be important and was felt by the senior surgeons to be, in part, a training issue.

It became clear that ‘performing procedures safely and effectively requires adherence to meticulous techniques and decision making on the part of the surgeon’ was a factor in one-third of cases. In two-thirds of cases, problems arose due to physiological changes in the patient such as periprosthetic fracture, or due to implant wear or reactivation of deep infection due to immune system failure. Also, surgery is well known to have an element of risk leading to unpredictable outcomes.^8^ A previous study based in Argentina has found an overall 90-day complication rate of 12.7%^11^; the most frequent complications were anaemia requiring blood transfusion (27%), wound infection (15.6%) and urinary tract infection (6%). A total of 23.2% required further intervention, and the mortality rate was 3.6%.

This study has shown a lower complication rate (1.45%) and a mortality rate of 0.76%, with rates of complication similar to those quoted in the literature for elective surgery outcomes for dislocation, periprosthetic fracture and infection.^13,28–30^ However, the aim of the paper is rather to continue to learn from even a single event through internal reflection and inspection of the themes and allocation of resources. The literature concerning the definitions, collection and interpretation of data regarding complications is often difficult to interpret. This causes problems in the comparison, analysis and improvement of surgical practice.^31^

In this study, every complication was considered a new event, although 14 patients had multiple complications. This balances out for patients with complications who have gone to other organisations. The total number of elective cases requiring reoperation was 374. The cases from different hospitals accounted for 41%. This shows that there is a considerable resource implication for managing complex care, some of which is inherited for problems that have arisen from surgery performed in other institutions. Currently there is no method for trusts to recoup the financial implications of carrying out this work.

Rates of surgical tourism in geographical regions or internationally are ill defined and this has been, to a certain extent, encouraged through the adoption of elective care centres.^32^

Some complications may be avoided by early intervention, such as hip replacement. Some of the decision making around this has been improved by having MDT meetings for failed hip and knee arthroplasty, especially due to perioprosthetic fracture, dislocation and infection. In a similar way, upper limb surgeons also discuss complex shoulder and elbow pathology at MDT. Finally, almost all patients have a MDT as part of the morning trauma meeting supported by subspecialists.

This study is limited in that it reports only those patients requiring readmission to our hospital under the care of the trauma and orthopaedic team. Readmissions to other hospitals or departments, for example, under medical physicians for urinary tract infections or thromboembolic disease and pressure ulcers reported only to the nursing staff were not included in this study as they were not captured by the local departmental database. However, it is departmental policy for any infection or complication relating to a previous surgical procedure to be cared for by that specialty team and not general medicine even if surgical treatment is not required and, therefore, we anticipate a low number of missed cases.

No data from national databases or registry data were sourced because the main objective was to derive learning to reduce the risk of adverse events and share the processes that help with that reflection. Intraoperative complications were recorded and discussed but accounted for a very small number (37 cases in this study; 8 vascular injuries, 7 nerve injuries and technical issues with surgery including 14 femoral calcar fractures). This relied on the surgeons self-reporting intraoperative difficulties and was prone to reporting bias. Patients re-presenting and captured by the department through the admission records were therefore detectable independently. Some intraoperative complications were reported through incident reporting and a risk meeting or in the subspecialty meetings. To address this concern and improve the robustness of surgical delivery, the department introduced a weekly x-ray review meeting of cases led by the fellows and specialty doctors in the department to derive learning. This is an area where improved surveillance is needed and is a limitation of the study.

Sharing readmission data between specialties is important for safer systems of working and is encouraged in the NHS, especially as complex care through readmissions leads to increased costs, morbidity and mortality. No local hospitals have a system that specifically tracks all the readmissions in orthopaedics, that the authors are aware of, that has fed back to other local hospitals. The hospitals share MDT meetings where cases are discussed and flow back, but the ability to monitor complications regionally and feed into local morbidity and mortality meetings would be a recommendation.

Consideration of how surgeons train and maintain competency is important as this impacts on care and adverse outcomes, as seen recently with the increased adverse events in surgeons undertaking fewer shoulder replacements in the UK.^33^ There are well-reported learning curves for different procedures such as peri-acetabular osteotomy, where major surgical complications are not uncommon within the first 20 cases.^3^ Altering the surgeon’s technique can mitigate some risk in this procedure by using a double incision technique that extends the operating time, which may make the surgery more physically and mentally demanding.^3^ More recently, there has been advice for low-volume and high-complexity procedures to be performed by dual consultant surgeons, for example in elbow arthroplasty and paediatric surgery, to reduce complications and improve patient outcomes.^34^

The role of audit and multidisciplinary meetings in learning from previous experiences and shared wisdom is clear and started when managing patients with cancer diagnoses.^35^ Multidisciplinary meetings are a daily occurrence in orthopaedics to help manage trauma, hip fracture, spinal injuries and now as review meetings to look at outcomes in arthroplasty surgery.^36–38^ The question arises as to how to drive learning that informs practice from audit meetings in surgery in general, and part of this may be to use a critical lens such as now used in mortality reviews in most NHS trusts. Similar processes are now to be encouraged when reviewing the outcomes of complications. Most units and regions now work as part of MDTs that review revision procedures, infections and discuss complex cases. These discussions in groups help crystallise the learning in subspecialties for wider dissemination among colleagues to improve safety.
